# Beyond Average: α-Particle Distribution and Dose Heterogeneity in Bone Metastatic Prostate Cancer

**DOI:** 10.2967/jnumed.123.266571

**Published:** 2024-02

**Authors:** Nadia Benabdallah, Peng Lu, Diane S. Abou, Hanwen Zhang, David Ulmert, Robert F. Hobbs, Hiram A. Gay, Brian W. Simons, Muhammad A. Saeed, Buck E. Rogers, Abhinav K. Jha, Yuan-Chuan Tai, Christopher D. Malone, Joseph E. Ippolito, Jeff Michalski, Jack W. Jennings, Brian C. Baumann, Russell K. Pachynski, Daniel L.J. Thorek

**Affiliations:** 1 Mallinckrodt Institute of Radiology, Washington University in St. Louis School of Medicine, St. Louis, Missouri;; 2 Department of Biomedical Engineering, Washington University in St. Louis, St. Louis, Missouri;; 3 Department of Molecular and Medical Pharmacology, UCLA, Los Angeles, California;; 4 Division of Oncology and Pathology, Department of Clinical Sciences, Lund University, Lund, Sweden;; 5 Department of Radiation Oncology, Johns Hopkins University, Baltimore, Maryland;; 6 Department of Radiation Oncology, Washington University in St. Louis School of Medicine, St. Louis, Missouri;; 7 Center for Comparative Medicine, Baylor University, Houston, Texas;; 8 Department of Medicine, Washington University in St. Louis School of Medicine, St. Louis, Missouri;; 9 Department of Radiation Oncology, Springfield Clinic, Springfield, Illinois; and; 10 Oncologic Imaging Program, Siteman Cancer Center, Washington University in St. Louis School of Medicine, St. Louis, Missouri

**Keywords:** α-particle, dosimetry, ^223^Ra, biopsy, autoradiography

## Abstract

α-particle emitters are emerging as a potent modality for disseminated cancer therapy because of their high linear energy transfer and localized absorbed dose profile. Despite great interest and pharmaceutical development, there is scant information on the distribution of these agents at the scale of the α-particle pathlength. We sought to determine the distribution of clinically approved [^223^Ra]RaCl_2_ in bone metastatic castration-resistant prostate cancer at this resolution, for the first time to our knowledge, to inform activity distribution and dose at the near-cell scale. **Methods:** Biopsy specimens and blood were collected from 7 patients 24 h after administration. ^223^Ra activity in each sample was recorded, and the microstructure of biopsy specimens was analyzed by micro-CT. Quantitative autoradiography and histopathology were segmented and registered with an automated procedure. Activity distributions by tissue compartment and dosimetry calculations based on the MIRD formalism were performed. **Results:** We revealed the activity distribution differences across and within patient samples at the macro- and microscopic scales. Microdistribution analysis confirmed localized high-activity regions in a background of low-activity tissue. We evaluated heterogeneous α-particle emission distribution concentrated at bone–tissue interfaces and calculated spatially nonuniform absorbed-dose profiles. **Conclusion:** Primary patient data of radiopharmaceutical therapy distribution at the small scale revealed that ^223^Ra uptake is nonuniform. Dose estimates present both opportunities and challenges to enhance patient outcomes and are a first step toward personalized treatment approaches and improved understanding of α-particle radiopharmaceutical therapies.

## Visual Abstract

**Figure:**
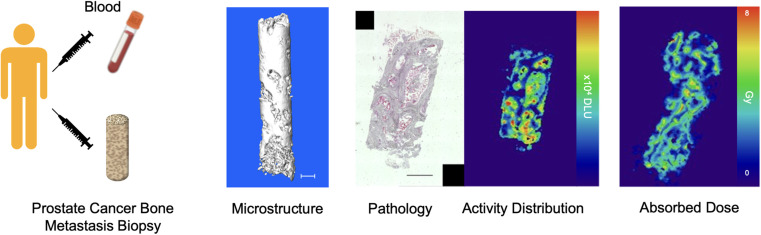

Prostate cancer is the second most frequently diagnosed malignancy in men, and an estimated 30,000 men were projected to die of the disease in the United States in 2023 alone (1*,*^2^). Early treatment for localized disease can be curative; however, locally advanced and disseminated prostate cancer is incurable. Bone metastatic castration-resistant prostate cancer (bmCRPC) is a frequent form of late-stage disease that is challenging to manage. Recently approved adoptive cell therapy, taxanes, DNA repair, and novel androgen-receptor-axis inhibitors have limited effects on bone lesions, which are associated with decreased survival and difficult-to-palliate pain (3).

α-particle radiopharmaceutical therapy (α-RPT), delivering megaelectronvolt energies over only several cell diameters directly to sites of disease, has garnered intense academic and clinical interest (4*,*^5^). At the vanguard of this class of potent agents is ^223^Ra-dichloride ([^223^Ra]RaCl_2_ citrate [Xofigo; Bayer]), the first and only approved α-RPT (6). Studies demonstrate improved overall survival, increased time to the first skeleton-related event, and reduced symptomatic pain (7–12). Although volumetric effects are limited, the ablative impact must be viewed in the context of the disease stage for which the drug has been approved and is comparable with other therapeutic modalities. Without an understanding of the local activity profile, we can only speculate that modest efficacy may be due to insufficient local dose.

Conventional external-beam radiotherapy produces uniform absorbed-dose fields. These contrast with radiopharmaceutical therapy, which can accumulate heterogeneously at any site. However, current methods assume unrealistic uniform activity distributions, often informed by noninvasive imaging. [^223^Ra]RaCl_2_ distribution studies have focused on organ-scale pharmacokinetics using scintigraphy and emerging SPECT methodologies that are incapable of resolving α-RPT distribution at a cellular resolution (13–17). It is at these dimensions that the doses are deposited, resulting in uneven distributions that are relevant for both tumor effects and marrow toxicity for this bone-seeking ion (18*,*^19^). Clinical trials involving [^223^Ra]RaCl_2_ have revealed that average absorbed-dose estimates to the red marrow do not accurately predict suppression (20).

It is imperative to develop advanced methodologies that enable optimization of individual patient treatment plans while concurrently strengthening the robustness of radiobiologic studies. Small-scale characterization is necessary to understand the clinical effects of these potent radioactive emissions in healthy and diseased tissues. This can be used to precisely assess dose (21*,*^22^) and to guide optimized use of these potent therapies (23*,*^24^). There is a dearth of data at this scale, with no primary data on the activity distribution of α-RPT clinically needed to improve small-scale models or inform personalized treatment approaches (25–27).

To define the distribution and absorbed doses at bmCRPC sites, we acquired multimodal imaging and high-resolution quantitative ^223^Ra autoradiography of patient biopsy specimens. We observed heterogeneous distribution of the α-emitter, primarily localized at the bone–tissue interface within lesions, leading to spatially nonuniform absorbed-dose profiles. These data provide insight into the complex microstructure of pathologic bone metastases and variability in the magnitude and spatial distribution across patients and across lesions, providing a basis to measure effects of ^223^Ra and other investigational α-RPT.

## MATERIALS AND METHODS

### Patients and Biopsy

Patients diagnosed with bmCRPC were treated with [^223^Ra]RaCl_2_ citrate at the standard activity of 55 kBq/kg. Written informed consent was obtained for all patients (*n* = 7) under Institutional Review Board protocol 201411115. Patients received pretreatment ^99m^Tc-methyl diphosphonate bone scans, and candidate osseous lesions were identified. Blood samples were collected 24 h after therapy, followed by CT-guided percutaneous drill-assisted biopsy using the coaxial OnControl system (Teleflex Arrow).

### Sample Preparation and Counting

Samples were weighed on a microbalance (XP204; Mettler Toledo) and subsequently fixed (4% paraformaldehyde and 30% sucrose, each for 24 h). Biopsy and blood (triplicate, 2 mL) samples were γ-counted using an open-window protocol for 10 min using a National Institute of Standards and Technology source–calibrated system (Wizard^2^; Perkin Elmer) (28). Spectral acquisitions of biopsy specimens and pooled blood were conducted for 1 h on a high-purity germanium system (GEM-50195-S and Gamma-Vision version 8.0; Ametek). Samples were placed directly on the aluminum endcap, enclosed in a 10-cm lead shield (HPLBS1; Ametek). Finally, biopsy specimens were cryoembedded (optimal cutting temperature compound; Sakura Finetek) without decalcification, as per our previous radium-preserving protocols (29*,*^30^).

### Micro-CT

Optimal cutting temperature compound–embedded biopsy specimens were scanned by high-resolution micro-CT (VivaCT40; Scanco). Samples were secured in a cylindric insert with dry ice and scanned at an isotropic voxel size of 12.5 μm (70 kVp, 114 μA) and analyzed in Amira (version 5.3.3; Thermo Scientific).

### Microdistribution and Histology

Cryoembedded biopsy specimens were sectioned onto an adhesive support and affixed to 2.54 × 7.62 cm (1 × 3 in) glass slides (8 μm; CM1860; Leica), within 48 h of biopsy. Approximately 100 sections per biopsy specimen were exposed on storage phosphor for digital autoradiography (DAR; CyclonePlus; Perkin Elmer), coexposed with [^223^Ra]RaCl_2_ activity standards, read out at 600 dpi (approximately 40-μm resolution; OptiQuant version 5.0; PerkinElmer), and further processed in ImageJ (31). Subsequently, sections were stained with hematoxylin and eosin (H&E) and scanned with a ×10 objective (Eclipse Ti2; Nikon).

We built an image-processing pipeline to segment H&E acquisitions and register to DAR (Supplemental Fig. 1; supplemental materials are available at http://jnm.snmjournals.org). H&E images were converted to the International Commission on Illumination color space, and 4 statistical features (mean, SD, skewness, and kurtosis) were extracted from each channel from image subtiles (30). K-means clustering of features defined nonosseous tissues, and a deep convolutional neural network was trained from 20 manually defined sections to segment the bone surface. Coregistration was accomplished by downsampling H&E micrographs to DAR resolution, determining bounding boxes of each section, and performing an initial automated registration (including scaling, rotation, and translation), as described previously (30). A finer automated alignment was then conducted to maximize the mutual information between the images. We defined the bone–tissue interface as 50 μm within each compartment.

### Small-Scale Dosimetry

Absorbed-dose distribution was assessed according to MIRD methodology using *D* = *A˜* × Δ × ϕ/mass, where *A˜* is cumulated activity, Δ is mean α-energy, and ϕ is the absorbed fraction, with extrapolation to infinity, yielding a maximally conservative estimate (32). Activity in each voxel was calibrated using coimaged standards. Activity was decay-corrected to the time of biopsy. Finally, *A˜* was calculated by assuming that ^223^Ra and its daughters (^219^Rn, ^215^Po, and ^211^Bi) were fixed in the bone (18), that all decays occurred within the same voxel, and that all α-energy is deposited locally (ϕ = 1) with a voxel size of 43.2 × 43.2 × 8 μm. Energy values were sourced from International Commission on Radiological Protection publication 107 (33), yielding Δ = 4.23 × 10^−12^ J/(Bq⋅s) for ^223^Ra and its daughters. The density of bone was set to 1.92 g/cm^3^ and that of soft tissue and the bone–tissue interface to 1.03 g/cm^3^ (34).

### Analyses and Statistics

Several figures of merit were selected from the accumulated activity distribution and dose information. For each section, activity per voxel per tissue compartment was determined. Representative DAR and fusion of the tissue compartment and dose-map images are reported in digital light units or voxel dose values (Gy), respectively. Interactive Data Language (version 8.7.2; Harris Geospatial Solutions, Inc.), MATLAB (version R2016B; MathWorks), and Prism (version 10; GraphPad) were used for computations and statistical analyses.

## RESULTS

### Macrodistribution

**FIGURE 1.**
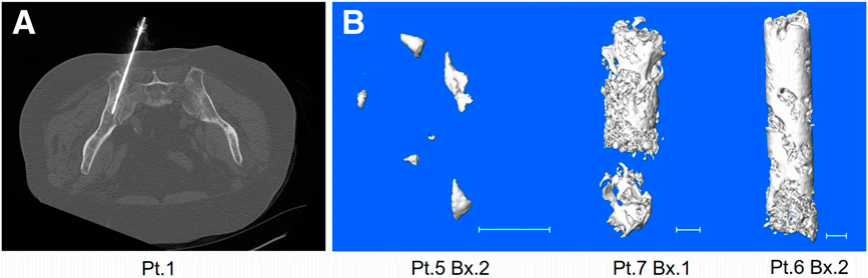
(A) Axial slice at collection from CT-guided biopsy of representative patient. Sample collection and biopsy needle location are shown entering ilium. (B) Micro-CT of biopsy (Bx.) 2 of patient (Pt.) 5, Bx. 1 of Pt. 7, and Bx. 2 of Pt. 6 (from left to right). Scale bar is 1 mm.

The present study involved 7 patients with bmCRPC treated with [^223^Ra]RaCl_2_. Men with a median age of 70 y (range, 66–78 y) and weight of 102 kg (range, 70–138 kg) underwent standard-of-care biopsy 24 h after α-RPT, with individual patient characteristics presented in Table 1. Prior bone scans were used to identify regions of active bone remodeling indicating a lesion, and both appendicular and axial skeletal sites were sampled (Fig. 1A).

**TABLE 1.** Patient and Biopsy Information

Pt.	Age (y)	Weight (kg)	Bx. at fraction	Activity injected (MBq)	Biopsy site	Number of cores
1	70	104	4	5.7	R ilium	2
2	70	117	2	6.5	L ilium	6
3	78	81	1	4.6	T12 vertebra	2
4	66	102	1	5.8	L5 vertebra	2
5	69	138	1	7.8	R humeral head	2
6	67	86	1	4.8	R ilium	2
7	73	70	2	3.9	L2 vertebra	1

Pt. = patient; Bx. = biopsy.

**FIGURE 2.**
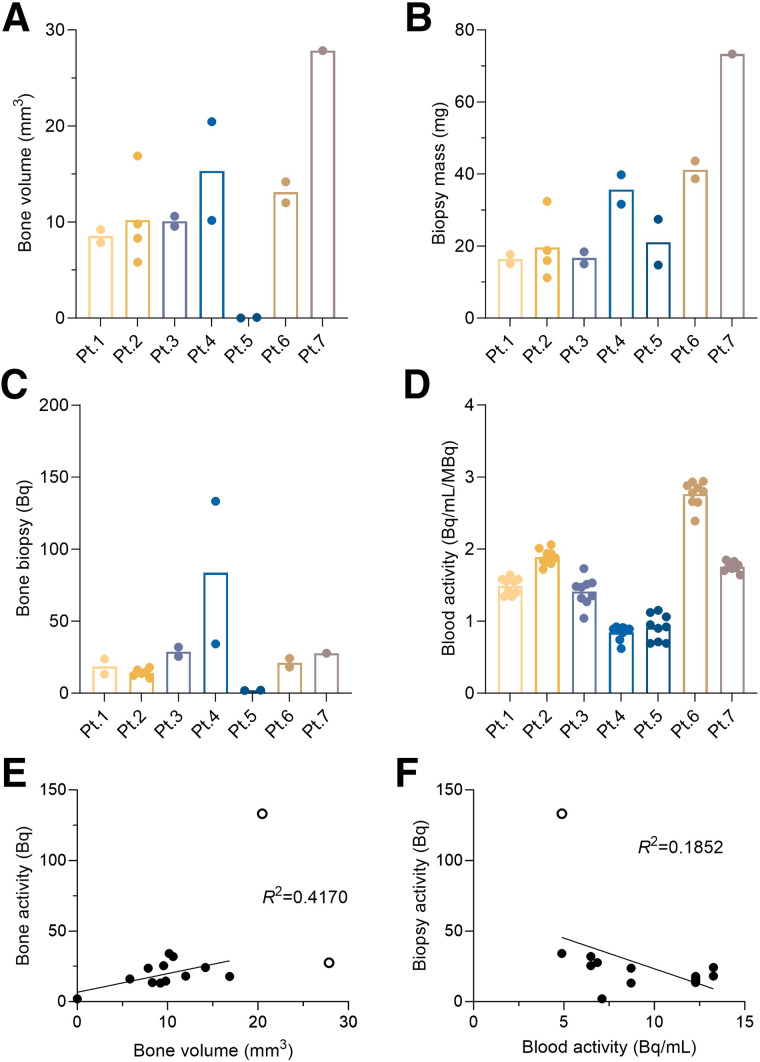
(A and B) Bone volume from micro-CT scan (A) and mass of each biopsy specimen (B). Mass of biopsy specimens from patients 1 and 2 was estimated using bone and tissue volumes measured from micro-CT. (C) Activity of ^223^Ra in biopsy specimens for each patient. (D) Concentration of ^223^Ra in blood samples for each patient’s normalized to administered activity. (E and F) Correlation of biopsy activity and bone volume (E) and blood activity (F). Open circles in E and F are outliers removed from correlation. Pt. = patient.

Bone content at these pathologic sites varied, as seen in volume-rendered high-resolution micro-CT (Fig. 1B). These scans were used to measure the bone volume present in each biopsy specimen (Fig. 2A). Typically, the pathologic biopsy specimen dimensions were 2 mm in diameter by 7 mm in length. The cores were composed of a mixture of cancer cells, soft tissue, and bone compartments, reflected in the differences in bone volume and microbalance measures of total biopsy specimen mass (Figs. 2A and 2B). Bone volume and mass of biopsy specimen from patient 5 (0.07 mm^3^ and 14.7 mg, respectively) and patient 7 (27.8 mm^3^ and 73.3 mg, respectively) represent the bounds of the bone-volume range sampled.

Activity in each biopsy specimen was assessed by γ-counting and verified by high-purity germanium (Supplemental Fig. 2). There is significant variability in the activity per sample at the subkilobecquerel level across patients and across samples (Fig. 2C). Biopsy specimen activities range from the limit of detection (0.0037 Bq) to 133 Bq. Blood samples were also collected before the biopsy. We observed circulating activity at 24 h of 5–13 Bq/mL and of 1–3 Bq/mL/MBq, when normalized to the administered activity (Fig. 2D). High-purity germanium γ-spectroscopy revealed that parent ^223^Ra was detected at or near secular equilibrium (measured <4 h after venipuncture; Supplemental Fig. 3).

A weak Pearson correlation coefficient between biopsy specimen activity and bone volume can be distinguished, despite the sublesion sampling (*n* = 12, Fig. 2E). By contrast, no clear correlation between the biopsy specimen and blood activity levels was discerned (Fig. 2F). Across samples, activity concentrations ranged from 0 to 27.1 Bq/mm^3^, with a median value of 1.7 Bq/mm^3^. Data for each biopsy specimen are included in Supplemental Figure 4, along with the bone biopsy specimen activity normalized to administered activity, which averages nearly 0.6 Bq/mm^3^/MBq. The individual blood concentration values (nonnormalized) are included for completeness (Supplemental Fig. 4C).

### Microdistribution

**FIGURE 3.**
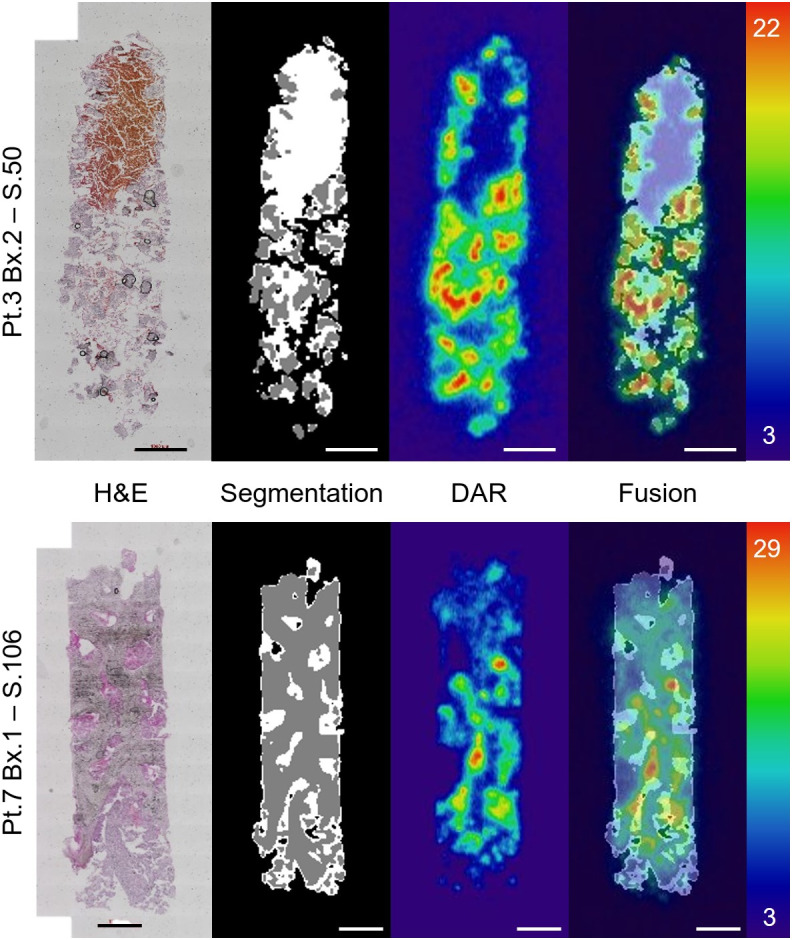
Representative workflow of 1 section from patient (Pt.) 3 (top) and Pt. 7 (bottom). From left to right, H&E acquisition; segmented compartments of soft tissue (white), bone (gray), and background (black); registered DAR; and fused result with autoradiography of sections matching. Scale is in digital light units × 10^4^. Training, segmentation, and autoregistration workflow schema are explained in Supplemental Figure 1. Bx. = biopsy; S. = section.

We next sought to determine the activity distribution within each biopsy specimen using an undecalcified sectioning technique and DAR (29*,*^30^). Approximately 100 sections were acquired for each sample along with a quantitation standard (DAR of first 42 sections of patient 3, biopsy 2 as an example dataset; Supplemental Fig. 5). Representative DAR and H&E micrographs are shown in Figure 3. H&E was used to define 3 compartments for bone, soft tissue (including marrow and prostate cancer), and the bone–tissue interface. Using a manually defined training set, we used an automated delineation of bone and soft-tissue compartments to segment the histologic data and coregister with the spatial distribution of radioactivity (Supplemental Fig. 1). The bone–tissue interface compartment was established as the boundary between the bone and the soft tissue and masks, and coregistered images are shown in Figure 3.

Fused high-resolution imaging reveals several features of the activity distribution near the cell scale. There is focused uptake of ^223^Ra along the bone–tissue interface, with nonuniform labeling displaying areas of both hot regions and no activity (Fig. 3). Intensity of the uptake decreases with distance from the bone–tissue interface. An extremely low signal was measured in the sections from the biopsy specimen of patient 5. Indeed, the biopsy specimen contains minimal calcified material (Fig. 1B; Fig. 2) and insignificant activity (Supplemental Fig. 6).

**FIGURE 4.**
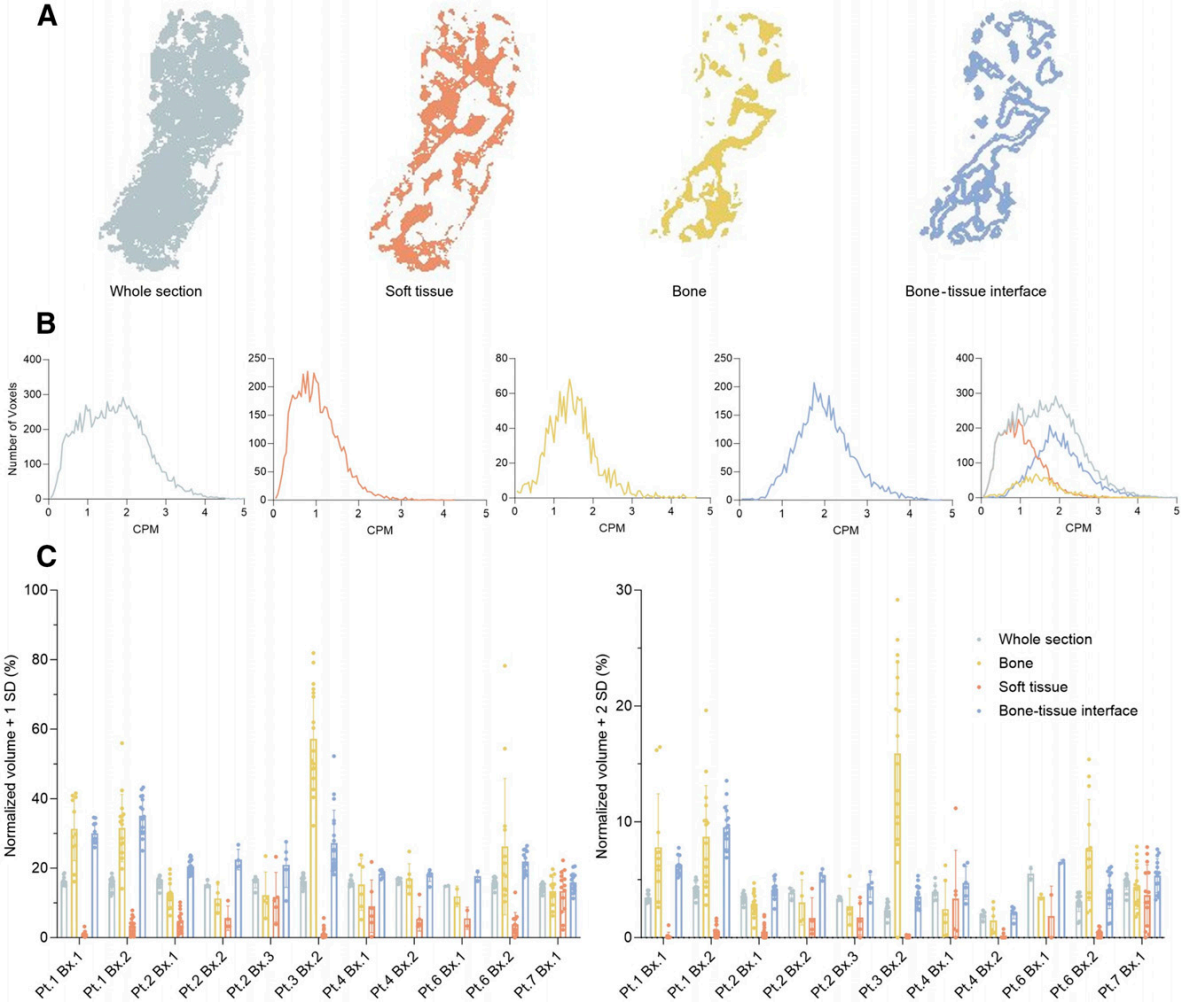
(A) Representative compartments for analysis, using section 77 of patient (Pt.) 1, biopsy (Bx.) 1, for soft tissue, bone, and bone–tissue interface. (B) Histogram of CPM measured in each compartment for same section. (C) Volume of each compartment over mean + 1 or 2 SDs for each patient biopsy. CPM = counts per minute; STD = SD.

To assess patterns of distribution, we determined the spatial activity profiles for each defined region across all evaluated biopsy sections. A representative compartmentalization of the whole section and segmented compartments is included (Fig. 4A), along with activity histograms for each (Fig. 4B). These data were normalized as volume per 8-μm section in tranches of first or second SD from the mean activity to evaluate heterogeneity of the ^223^Ra distribution (Fig. 4C). Bone and the bone–tissue interface are the regions that are distinguished as being above the mean. Soft-tissue regions consistently have the lowest activity profile and lack hot spots. Across the sections from a biopsy specimen, separated by hundreds of micrometers in depth, there is a clustering of the activity distribution profile; however, variability in these sample-normalized quantifications remains large.

### Small-Scale Dosimetry

**FIGURE 5.**
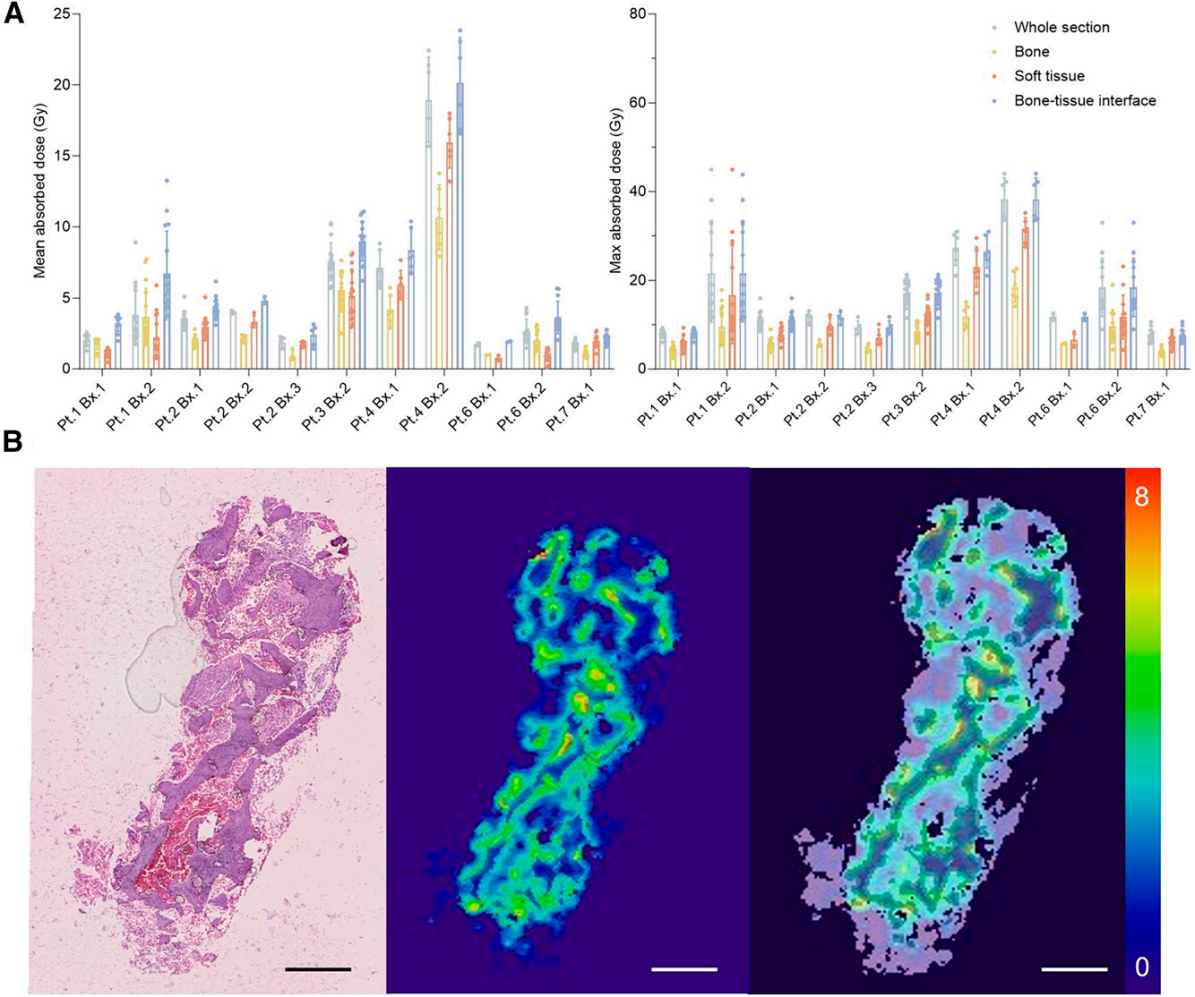
(A) Mean and maximum absorbed dose to compartment from direct measures of ^223^Ra distribution by patient and biopsy section (*n* ≥ 8) at left and right, respectively. (B) Representative biopsy specimen from patient 1 including H&E staining, computed absorbed dose distribution, and fused dose to compartment model segmentation. Scale is in Gy. Pt. = patient; Bx. = biopsy.

We next undertook novel measures of the absorbed dose at the small scale from these patient samples. Calibrated activity per voxel values from the sampled tissues at 24 h was used with the assumption that ^223^Ra localized to the bone and daughters decayed in place (18*,*^29^). We report mean and maximum absorbed-dose values for representative sections across patient biopsy specimens for whole samples and compartmentalized regions of bone, soft tissue, and the bone–tissue interface (Figs. 5A and 5B).

The highest absorbed dose values correlate with regions of greatest ^223^Ra localization, namely the bone–tissue interface. Values varied across patient samples in a range from 20.1 ± 3.2 to 1.9 ± 0.1 Gy at this surface (Supplemental Table 1). Modest differences between the mean absorbed doses measured in the bone–tissue interface were observed across sections of the same biopsy specimen, suggesting consistency within a bone metastatic core sample. Predictably, maximum absorbed-dose values have a greater range from 38.3 ± 4.8 to 7.5 ± 1.5 Gy at the surface (Supplemental Table 2). Most maximum dose voxels were found within this bone–tissue interface compartment, and thus maximum values of the whole section and bone surface values are concordant.

## DISCUSSION

The general approach of using mean activity concentrations to compute cumulated activity of a radiopharmaceutical as input for dosimetry calculations does not reflect the reality of highly localized distribution. Although adequate for γ-emitters in many contexts, these are insufficient to capture absorbed-dose profiles from spatially circumscribed interactions of α- and β-particle emitters at tens of micrometers and millimeters, respectively. The nonuniform cellular distribution of target cells and of ^223^Ra uptake and irradiation complicates the interpretation of the macroscopically averaged absorbed dose in terms of the biologic effect, and similarly, there are as yet no definitive evidence-based values for the relative biological effects of this therapy. [^223^Ra]RaCl_2_-treated biopsy specimens collected and analyzed here provide novel insight into α-RPT in metastases and to existing data of β-particle distribution in renal tissues and hepatocellular carcinoma (21*,*^22^*,*^35^).

Prior preclinical work has shown foci of ^223^Ra at sites of active bone turnover (29*,*^36^). Most ^223^Ra was localized to the bone–tissue interface in these clinical specimens, confirming this pattern of uptake at pathologic sites and underlining the role of lesional bone structure in activity distribution. Bulk measures of activity per mass of core, an accurate assessment of activity concentration, do not reflect the complexity of the activity or dose-distribution profiles. Mean absorbed-dose estimates vary from more than 20 Gy to (ostensibly) 0 Gy across bone surfaces of the samples (Fig. 5). With the caveat that we have sampled lesions rather than marrow sites specifically, this variability in primary data helps to substantiate the good safety profile with mild reversible myelosuppression despite model-based study estimates of endosteal cell and marrow mean doses of 16 and 1.5 Gy, respectively (18*,*^37^). This is, in turn, consistent with and confirms predictions from small-scale marrow modeling (25). Indeed, the results of this study can be implemented in bone-marrow models (2*,*^3^). Furthermore, dosimetry estimates based on patient-specific data could guide the optimal administered activity and personalization of [^223^Ra]RaCl_2_ therapy.

Absorbed-dose values in high-activity regions would be sufficient to ablate most metastatic and supporting cell types for many of the samples, considering the relative damage done by high linear energy transfer radiation. The computed values are for a single administration of [^223^Ra]RaCl_2_ (approved for use in 4 cycles separated by 6 wk). The information from the activity distribution and structural data suggests that for some metastases, a reduced number of treatments may be sufficient to control disease sites and that a subset of metastases will not benefit from either increased administered activities or increased cycles.

Histomorphometry is a core methodology for structure–function analyses in orthopedics; however, abnormal and diseased bone sites are rarely evaluated at this resolution. This is an interesting yet understudied area for which these samples provide additional value. The novel structural (micro-CT and H&E) and activity distribution (DAR) data collected here can further be used to inform small-scale dosimetry, essential to improving our understanding of anticancer and normal-tissue effects (38*,*^39^).

Nuclear and anatomic imaging localized sites for CT-guided sampling. The low bone content and absence of detectable activity in patient 5 at both macro- and microscopic scales do not imply a low-quality biopsy but rather underline the tissue complexity found at sites of bmCRPC. Biopsy provides a subsample of the lesion, which may reflect only the tissue directly collected and not the greater lesion, let alone the total patient burden. Further, anatomic location, structural features, and prior therapy to bmCRPC sites may influence local uptake and global response (40), directions of future research. Advances in high-resolution imaging, Monte Carlo dose modeling, and measures of biologic impact can also be used to guide optimal use of α-RPT with the presented data (25*,*^30^*,*^41^).

We have demonstrated high-resolution α-distribution in affected tissues at the length scale of the interactions within clinical samples. Our studies confirm highly localized uptake of ^223^Ra across samples from multiple skeletal sites of metastasis, with a low-activity background in adjacent soft tissue. Although onerous, direct assessment distribution can be implemented to estimate the dose within days after sampling, and implementation of such data for treatment decision-making may be feasible in the future. Limitations of this study include cohort size and the fundamental limitation that a site can only be biopsied once. We also assumed that ^223^Ra and its daughters are fixed from the 24-h time-point measurement and have assumed disintegrations and α-particle deposition occur within single voxels. To further our understanding of cellular-scale effects of clinical α-RPT, samples from a wider cohort are being assembled, with rapid processing to determine the potential for local diffusion of daughters and with cell-scale multiomics to link the dose with tumor and marrow responses.

## CONCLUSION

We addressed the lack of primary information of α-RPT activity distribution at the scale of its effect via high-throughput evaluation of bone-lesion specimens. These results provide the first patient sample small-scale values for ^223^Ra. Highly nonuniform distribution and absorbed dose present opportunities and challenges to improved outcomes for patients receiving α-RPT. Further, these data serve as a benchmark for comparison with other bone-lesion–targeted and molecular radiotherapies.

## DISCLOSURE

This work was supported by NCI R01CA201035, R01CA229893, and R01CA240711 (to Daniel Thorek) and R01EB031962 (to Abhinav Jha). The Siteman Cancer Center is funded by NCI Cancer Center Support Grant P30CA091842. We thank the Washington University Musculoskeletal Research Center (P30AR074992) for micro-CT expertise. No other potential conflict of interest relevant to this article was reported.

## Notes

KEY POINTS **QUESTION:** Can we improve our understanding and application of α-particle emitter therapies by learning small-scale distribution and absorbed dose from patient samples? **PERTINENT FINDINGS:** In a cohort of 7 patients with bmCRPC treated with [^223^Ra]RaCl_2_ citrate, we evaluated the distribution of ^223^Ra in primary bone biopsy specimens. Autoradiography and histology were analyzed to reveal predominant localization at the bone–tissue interface in clinical specimens. Computed mean absorbed-dose estimates ranged from more than 20 Gy to 0 Gy, displaying distribution differences across and within patient samples. **IMPLICATIONS FOR PATIENT CARE:** This study highlights that macroscopic measurements inadequately capture the intricacies of lesion-scale activity distribution, establishing a foundation for enhancing treatment response through the development of more realistic dosimetry models.
